# Practice change in community health centers: A qualitative study of leadership attributes

**DOI:** 10.3389/frhs.2022.934688

**Published:** 2022-09-07

**Authors:** Jennifer H. Tektiridis, Natalia I. Heredia, Robert O. Morgan, Osama I. Mikhail, Betsy C. Risendal, Michelle C. Kegler, Maria E. Fernandez

**Affiliations:** ^1^The University of Texas MD Anderson Cancer Center, Houston, TX, United States; ^2^Center for Health Promotion and Prevention Research, School of Public Health, The University of Texas Health Science Center at Houston, Houston, TX, United States; ^3^School of Public Health, The University of Texas Health Science Center at Houston, Houston, TX, United States; ^4^Colorado School of Public Health, Anschutz Medical Campus, Aurora, CO, United States; ^5^Rollins School of Public Health, Emory University, Atlanta, GA, United States

**Keywords:** evidence-based practice, leadership, community health centers, qualitative research and analysis, full-range leadership theory

## Abstract

**Introduction:**

This paper explores leadership attributes important for practice change in community health centers (CHCs) and assesses attributes' fit with the Full-Range Leadership Theory (FRLT).

**Methods:**

We conducted four focus groups and 15 in-depth interviews with 48 CHC leaders from several U.S. states using a modified appreciative inquiry approach. Thematic analysis was used to review transcripts for leadership concepts and code with *a priori* FRLT-derived and inductive codes.

**Results:**

CHC leaders most often noted attributes associated with transformational leadership as essential for practice change. Important attributes included emphasizing a collective sense of mission and a compelling, achievable vision; expressing enthusiasm about what needs to be done; and appealing to employees' analytical reasoning and challenging others to think creatively to problem solve. Few expressions of leadership fit with the transactional typology, though some did mention active vigilance to ensure standards are met, clarifying role and task requirements, and rewarding followers. Passive-avoidant attributes were rarely mentioned.

**Conclusions:**

Our results enhance understanding of leadership attributes supportive of successful practice change in CHCs.

## Background

The increasing pace of discovery and development of evidence-based programs and practices (EBPs) and the delay in moving these practices into health care delivery settings is widening the gap between what we know and how we care for patients (1, 2). This increases pressure on health care leaders to successfully lead clinical practice change, that is, the uptake of effective health services in clinical practice in their organizations (3). Increasingly, discoveries in implementation science, a field which investigates methods of implementing EBPs to improve the quality of health services, have helped define the many factors influencing successful clinical practice change. Leadership, defined here as the process by which an individual influences employees and other critical stakeholders to achieve a shared goal (4), is one of these factors. Leadership influences the successful implementation of EBPs in several ways, including attitudes, engagement, commitment, involvement, and accountability, among others (5–9). Leadership also influences the organization or context in which the practice change occurs because leaders have a major role in addressing organizational factors such as priorities and resource availability (10–12). This role may precede (13), be a starting point for (14), or occur throughout the implementation of practice change (15). In several theories, models, and frameworks, leaders emerge at all levels of the organization and may include individuals in formal and informal leadership roles (5, 10). Researchers have highlighted the importance of context in implementation research (16–18). This can include the setting—such as the public health sector or clinical discipline—such as mental health (19), the interaction of leadership and organizational climate in health systems change (20), and the complexity of organizational processes (including leadership) and their effect on EBP implementation (21).

Community health centers (CHCs) provide a unique context because they are safety net primary care settings that serve a medically uninsured or underinsured population, receive significant federal funding, and have an important role in health care delivery (22). Because they serve those with a limited ability to pay for health services, these centers experience substantial resource constraints and are highly dependent on government funding requiring achievement of evidence-based clinical service delivery metrics (23) (e.g., tobacco cessation counseling, Papanicolaou tests for cervical cancer, and fecal occult blood tests for colorectal cancer). The clinical services measures and goals change periodically, requiring health care professionals in CHCs to modify their practices to achieve targets and making the ability to effectively implement practice change a key factor in sustaining CHC funding.

Because leadership is important to effective practice change, CHC leaders must understand the specific skills required to effectively lead. While implementation science models and frameworks suggest the importance of leadership, especially first-level leaders, what defines effective leadership practice is not fully discernible from existing models (24, 25). Further understanding what characterizes leadership in the context of practice change in CHCs will advance the field of implementation science and contribute to more effective practices to strengthen health services delivery in CHCs. Full-Range Leadership Theory (FRLT) is a widely used and enduring leadership theory with a validated measurement instrument (26, 27). The FRLT organizes leadership attributes into 3 typologies—transformational, transactional, and passive-avoidant—and describes 9 leadership factors associated with each (Table 1) (28). FRLT assumes that every leader employs each of the FRLT typologies to varying degrees and that one style may be more effective in certain circumstances than in others (29).

**Table 1:** Full-range leadership theory typologies, factors, and attributes.

**Typology**	**Factor**	**Attribute**
*Transformational Leadership* proactive, encourages followers to push toward higher standards, motivates teams to achieve both the leader's and team members' goals, and directs priorities toward creativity and innovation	Idealized Influence (attributed) Leaders earn the trust and respect of followers by acting in the interests of the group and organization, focusing on higher order ideals, and emphasizing a collective sense of mission (i.e., leaders use power and influence not for personal gain but to advance the organization toward its vision).	**Is focused on higher order ideals**
		Is perceived as powerful and confident
		Ethical
		Charismatic
	Idealized Influence (behaviors) Same as above, but this is the team members' observations of and responses to leader behavior and actions.	**Emphasizes a collective sense of mission**
		Has a sense of mission/purpose
		Acts based on values
		Acts based on beliefs
	Inspirational Motivation Leaders articulate a compelling future vision that engages followers in a way that provides meaning to their work and enlists their participation in envisioning future states, ultimately generating a team spirit, and enthusiasm and optimism for achieving the mission.	**Communicates that the vision is achievable**
		**Expresses enthusiasm about what needs to be done**
		**Articulates a compelling vision of the future**
		Expresses optimism about the future
	Intellectual Stimulation Leaders inspire and stimulate the development of new ideas from team members and help them think about problems in new and creative ways. They encourage team members to question their own and others' assumptions and thus they develop team members' capacity for problem solving.	**Challenges team members to find solutions to difficult problems**
		**Appeals to team members' analytical reasoning**
		Appeals to team members' sense of logic
		**Challenges team members to think creatively**
	Individualized Consideration Leaders pay attention to team members as individuals in a coaching role with the aim of developing the individual to achieve higher levels of performance.	Pays attention to individuals
		Advises on the basis of individual needs
		Supports individuals
*Transactional Leadership* provides rewards contingent on performance and goal achievement and takes corrective action when team members do not meet established expectations	Contingent Reward Leaders assign or gain concurrence from team members to carry out an assignment. If the team members accomplishes the assignment, the result is rewarded.	**Clarifies role and task requirements**
		**Provides team members with contingent material or psychological rewards**
	Management by Exception (active) Leaders see their role as exchanging value (rewards and incentives) for team members actions (achieving performance levels), with a focus on problems, often described by metrics that fall below a defined target. Errors, deviations from standards, and failures to achieve results are corrected to achieve results are corrected.	**Uses active vigilance to ensure standards are achieved and goals are met**
*Passive-Avoidant Leadership* avoids and abdicates leadership responsibilities, is reactive, and does not establish standards or goals for team members	Management by Exception (passive) Leader does not set expectations or monitor results. When errors occur, the leader waits until problems are chronic and serious before taking action.	Intervenes after mistakes are made
	Laissez-Faire Leader avoids leadership, is indecisive, delays actions, and ignores their responsibilities as a leader.	Avoids making decisions
		Chooses not to take action
		Abdicates responsibility
		Does not use authority

Bold indicates FRLT attributes that were most frequently expressed by participants in this current study. Adapted Table (20).

In this qualitative study, we explored what FRLT leadership attributes CHC leaders thought were important in their role of supporting practice change in CHCs. A qualitative assessment of these attributes can provide a more robust, in-depth understanding on the attributes that would be missed with quantitative research.

## Methods

### Study design

This was a secondary analysis of data collected through the Cancer Prevention and Control Research Network (CPCRN), a Centers for Disease Control and Prevention–funded collaborative of academic centers tasked with accelerating EBP adoption for cancer prevention and screening. The parent study aimed to assess organizational factors influencing adoption of evidence-based cancer prevention and control studies in the CHC setting. This focus was selected because of the close alignment with the current environment in healthcare delivery that embraces continuous practice transformation. (30). Understanding the process of practice transformation through the lens of evidence-based cancer screening interventions can assist CHCs with transformation initiatives such as Achievement of Meaningful Use and Patient Centered Medical Home Recognition.

The parent study investigators collected data from 4 focus groups and 15 in-depth interviews using structured interview guides generally informed by the Consolidated Framework for Implementation Research (CFIR) (5). The focus group and interview guides included open-ended questions about practice change with cancer prevention and screening as the example, given that delivery of these clinical services was relevant and common to all CHCs in the sampling, and thus participants would be able to more readily connect to this specific practice change example. Trained interviewers used a modified appreciative inquiry approach to elicit participants' core motivations (31). As part of this approach, interviewers asked about successful practice change experiences to understand the strengths and values inspiring the participants and fostering positive relationships with colleagues and facilitators. They later asked about barriers to successful practice change.

**Figure 1:**
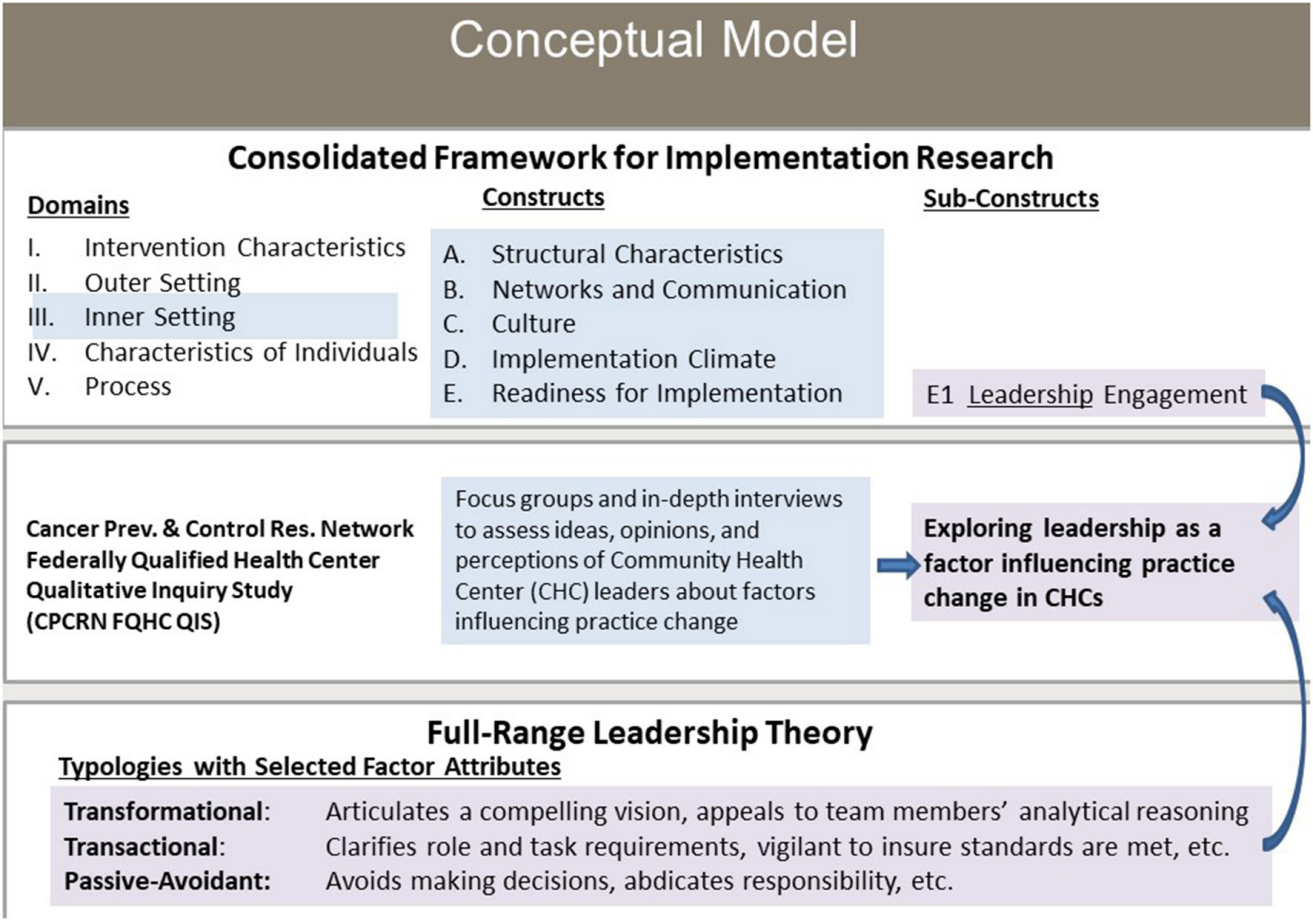
Conceptual model: exploring leadership as a factor influencing practice change in community health centers. CHC, community health center. FQHC, federally qualified health center.

The guides included a question regarding leadership. Specifically, participants were asked, “*How do you think the characteristics of the organization (for example, the size, values, leaderships [quality improvement] plan) might affect adoption or implementation of this strategy?”* with the follow-up question, “*Tell me about the leadership in your organization. Does the leadership encourage change?”* We did not distinguish between leadership and management in this study. This study's conceptual model depicts the link between implementation research theory, represented by CFIR, and leadership theory, with FRLT as the basis for the theory-driven applied thematic analysis (Figure 1).

### Study participants

Study staff recruited participants for the larger CPCRN Federally Qualified Health Center Qualitative Inquiry Subgroup through study investigators' partnerships with various federally qualified health centers and primary care organizations in 5 states and the District of Columbia. *Via* email, CPCRN investigators and staff invited attendees of the organizations' conferences and meetings (Table 2) to participate on-site at the conferences or during separate meetings with leaders and members of the partner organizations. A total of 48 individuals with leadership roles participated in the interviews and focus groups (Table 2).

**Table 2:** Focus group and interview details and participant demographics.

**Data Collection Activities**	**Date**	**Number of Interviews/Groups**	**Participant Gender**		**Participant Role**				
			Male	Female	CMOs/ Medical Directors	CEOs	COOs/ Operation Directors/ Managers	Other Leaders	**Total**
Focus group: National Association of Community Health Centers Community Health Institute (San Diego, CA)	Aug 29, 2011	1	2	2	4				4
Focus group: Colorado Primary Health Care Association Meeting (Denver, CO)	Mar 8, 2012	2	15	8	15		8		23
Interview: National Association of Community Health Centers Policy and Issues Forum (Washington, DC)	Mar 21–25, 2012	12	7	5	7	3	1	1^*^	12
Focus group: South Carolina Primary Health Care Association Quarterly Meeting (Columbia, SC)	Apr 8, 2012	1	2	4	2			4^y^	6
Interview: Georgia Association for Primary Health Care Patient Center Medical Home Learning Session (Pine Mountain, GA)	Apr 18–19, 2012	3	2	1	1	1		1^z^	3
Total		**19**	**28**	**20**	**29**	**4**	**9**	**6**	**48**

* Executive Vice President and Chief, Clinical Quality and Training.

y 2 Directors of Nursing (1 also noted Quality Services), 1 Nurse Supervisor, and 1 Director of Health Services.

z 1 Director of Nursing.

CEO, chief executive officer; CMO, chief medical officer; COO, chief operations officer.

### Data collection

Staff piloted the focus group guide with the first participant group and modified the guide as needed. Focus groups lasted 1–3 h and interviews lasted 40–90 min. The interviewers obtained written informed consent, recorded the focus groups and interviews, and took notes during or immediately after the sessions. Participants received $50 for participation. Staff de-identified transcripts and a transcription service transcribed the audio recordings. The Committee for the Protection of Human Subjects at the UT Health Science Center at Houston approved this study.

### Data analysis

We conducted a theory-driven applied thematic analysis using Atlas.ti to identify expressions of leadership for practice change. We did not find a distinction between leadership and management in our initial review of the data, supporting our decision to not make a distinction between these in this study.

We developed codes using a hybrid deductive and inductive process (32). JT coded text on the basis of the 27 unique attributes from the 3 FRLT leadership typologies (26, 28). while also looking for new concepts as part of inductive coding. Coding was done in several steps, with the first step being to read the transcripts, identify passages of text that contain expressions of leadership, then mark these as “quotations” (using Atlas.ti terminology). These “quotations” were coded to existing FRLT-driven codes at the attribute level or new codes were created. New codes were annotated with comments to provide additional detail as to the concepts the code was intended to capture. This facilitated the review of new codes to identify and consolidate codes that represented a same or similar (non-unique) leadership concept. After eight transcripts were coded, codes were reviewed by and discussed with MEF. At the end of the coding process, codes were reviewed to determine if they should be further consolidated based on three or fewer quotations per code. Codes with 15 or more quotations were reviewed to determine if they represented multiple concepts that were captured separately in other codes. Finally, the new codes were assessed for fit with the FRLT-based attribute codes and mapped to these. The 9 new codes that did not map to FRLT attributes were reviewed further and grouped into three new themes by JHT and MEF. We reviewed the network in Atlas.ti, including the number of codes and quotations linked to a factor's attributes, to identify any dominant leadership typologies or attributes within a typology.

## Results

Below we first describe the transformational leadership attributes, which participants expressed most frequently (Table 1). Then we describe transactional typology factor attributes, mentioned by some participants. We do not discuss passive-avoidant attributes, as they were not salient. Lastly, we describe the three new emergent themes.

### Transformational leadership: Inspirational motivation

Most frequently, participants mentioned transformational leadership attributes that fit with FRLT model attributes encompassed by inspirational motivation. This factor describes leaders who articulate a compelling future vision that engages followers by providing meaning to their work and enlisting their participation in envisioning future states, ultimately generating a team spirit and enthusiasm and optimism for achieving the mission. They also described a variety of messages used to communicate confidence in their employees' abilities to achieve practice change goals, including messages that first recognize the challenge of change and then appeal to employees' motivation to engage. Leaders described acting to signal their commitment to goal achievement while noting that energizing employees to act is a constant challenge. They articulated a need to advocate for initiatives, embrace change, understand enablers of practice change, make the case for change to their staff, and share experiences—good and bad—in making practice changes. One individual, describing making the case for practice change, said, “And once we started doing that with the entire staff, it was like, ‘wow, we are part of the bigger picture, they are going to compare us to the state and to the national'…. I put up some of the numbers that weren't as good. You know, they go ‘oh, what can we do?”' When leaders described their advocacy for initiatives, they emphasized the importance of conveying their enthusiasm for meeting patient needs, even if the catalyst for change was initially something else, such as achieving minimum levels of performance for government-mandated pay-for-performance measures such as those in the Uniform Data System (UDS) required for US CHCs.

Participants emphasized that those articulating a compelling vision of the future motivate and inspire employees and other stakeholders to act in ways that advance the organization's goals. Leadership activities they mentioned included sharing leadership's vision with their team by articulating the rationale for and benefits of change. Leaders described the importance of beginning practice change with a clear goal so that they could communicate that goal to employees and important stakeholders to inspire and facilitate their engagement. As one participant put it, “You need to know ahead of time what success is going to look like to you and make sure that as you're going through this you have a mechanism so that you're able to monitor and see how you're doing.”

### Transformational leadership: Intellectual stimulation

Leaders described the need to challenge members of their team to solve difficult problems and challenge the status quo. For example, one leader mentioned the importance of recognizing that the “idea that only the provider can do this is not really realistic because every single public health guideline seems to think that the provider can do it…we have other people who have training who I think are just as capable and maybe in some cases are better suited to do it.” They also described working collaboratively by integrating multiple stakeholder views and recommendations.

Leaders described the need to engage their team and external partner organizations in thinking creatively about how to make practice change responsive to external demands, often without sufficient resources. One individual emphasized the need to engage with others, stating that “The challenges are kind of taking a good idea and making it tangible and real…getting help from folks who know their pieces of it much better than the others do…and as usual, it's a process of drawing on the experiences and expertise of a lot of different folks.” They also described creative thinking as thinking strategically and applying past experiences to new situations.

Leaders expressed the need to appeal to team members' analytical reasoning, which includes relying on the evidence base in selecting interventions and thereby making data-driven decisions. For example, one individual said, “Particularly for the people who are being asked to do something different…you can always reinforce them and tell them you're really grateful and they're doing a nice job…but I think data is a lot stronger message.”

### Transformational leadership: Idealized influence (attributed)

When leaders described engagement within the organization and with external partners, they emphasized a collective sense of mission. Forgoing personal gain to act in the best interests of the CHC is one way they demonstrate their commitment to higher-order ideals, such as those described in the CHC's mission. As one said, “You know, without good leadership, there is no change.”

### Transformational leadership: Idealized influence (behaviors)

Leaders described appealing to employees as a team to embrace the mission, encouraging them to work together and align efforts to achieve goals. They noted the importance of reinforcing team orientation when recognizing goal achievement and the satisfaction expressed by the team about achieving its goals. One leader described the effect of a team's success on employees, “… we saw results and fed them back, people were so glad to be a part of a winning [team] and then when we got that award from the Health and Human Services …when we do the staff satisfaction surveys, we saw this great response …That's really what kind of motivated us on was that employee reaction.”

### Transactional leadership: Management-by-exception—active

Leaders described focusing on performance data and needing to meet externally imposed standards, such as UDS metrics and stated that electronic health records (EHRs) make issue identification easier, allowing for course correction when needed. While they acknowledged the need to be attentive in detecting and correcting problems, some noted that simply collecting performance data, and staff knowing that it is being collected, lead to performance improvements, “…and the other thing is if we monitor our results, I think that results will improve. There's no doubt. The old saying is what gets monitored gets done. So, if we measure our results, people will be thinking about it, and they will do something about it.” However, some leaders were frustrated when they could not obtain data from the EHR to monitor and communicate individual provider results, with one leader saying, “if 20 people are accountable for it and [it] doesn't work, which one of the 20 needs to change.”

### Transactional leadership: Contingent reward leadership

Leaders mentioned very few concepts representing the contingent reward factor. A few discussed providing their team members with contingent material or psychological rewards in the form of incentives and team member responses to the idea. Incentives can be based on various or multiple factors. One leader stated “We have an incentive program for our providers, and the measures on which the incentive is based are—volume is there, patient satisfaction is one of the measures, but then we also look at things like diabetes control, hypertension control, lipids for patients with diabetes.” Even when faced with limited funds and resources, one leader stated, in reference to an incentive program, that they were “still tossing this around … what we're going to do to reward people” suggesting that leaders recognize value in providing rewards contingent on achieving certain goals.

### Themes outside the FRLT model

We grouped 9 new codes that did not fit the FRLT model attributes into 3 themes: planning, seeking buy-in, and developing resources.

CHC leaders described the need to plan, generally in response to external forces that required additions or changes to clinical service delivery and affected reimbursement and payer incentives. In some cases, CHC partners, such as specialists to whom the CHCs referred patients, became unavailable, requiring leaders to make contingency plans for service. Forces prompting change also came from the community but were infrequently mentioned by the participants. Likewise, internal events (e.g., turnover or infrastructure issues) required a planning response. The need to make a plan including trade-offs to solve problems and prioritize clinic users' various needs was discussed. One leader asked, “… is it better to try to allocate more resources to help that person improve their diabetes, or do you allocate those same resources to do preventative cancer screenings?” Another planning dimension is sustaining previous improvements once priorities shift.

To advance the mission, leaders expressed the need to get buy-in from internal stakeholders (at all levels), external collaborators, partners, and key influencers, including the community, board of directors, and specialty care providers to whom the CHC may refer patients. In describing a new initiative, one participant said, “That was basically a combination of our management staff, it was our medical director, but it was also a buy-in by the executive director. There was a decision made to make this as a piece of business that we were interested in. So, we had to both put it on the clinical side and found interest in working on it, but on the administrative side they had to commit time and resources and have people work on it.”

The need for additional resources to implement practice change and, specifically, EBPs for cancer prevention and screening was also discussed. Leaders explained that competing priorities present challenges, especially when community needs must be addressed. They also talked about the need to be strategic when resources were limited. One participant described how partnerships and collaborations help bridge resource deficiencies.

## Discussion

Advancing preventive medicine requires understanding leadership in distinct settings. Therefore, our objective was to explore the leadership attributes CHC leaders considered important for practice change. We analyzed focus group and interview responses for fit with the FRLT model to determine the leadership typologies or factors dominant in CHCs. Participants described actions in the FRLT transformational typology but those in the transactional typology were few and those in the passive-avoidant typology were almost absent. However, new themes—planning, seeking buy-in, and developing resources—emerged.

The FRLT transformational typology was dominant. This aligns with previous studies, in which a transformational leadership style has been more associated with employee effectiveness and satisfaction (28), the innovation climate (19), and clinicians' attitudes toward adopting EBPs than have the transactional and passive-avoidant styles (19, 33). The CHC environment is complex (34) and serving vulnerable populations dependent on CHCs for their health care needs is always challenging, especially with limited resources (35). Thus, it is not surprising that leaders described actions taken to communicate a compelling, achievable vision and maintain enthusiasm for the mission. A leader's actions and words model the way forward and signal to their team members the leader's belief that the vision is achievable while also acknowledging the challenge of change. Perhaps because they are often called upon to collaborate internally and externally, leaders convey their commitment to higher-order ideals representing the CHC's mission and communicate these in multiple venues to seek support from individuals and entities that can advance that mission.

The optimal mix of FRLT attributes for leaders driving change has not been thought to be setting-specific (22, 36); however, emerging research is beginning to show that the setting may differentially influence the attributes most effective for a leader (37). It may be that the most effective mix of FRLT attributes is setting-specific and the FRLT is insufficient as a leadership model for CHC environments. Leaders in unique settings such as a CHC with a highly resource-constrained environment, serving patients with complex needs might need to expand their leadership behaviors beyond those defined by FRLT attributes to be effective.

Given funders' and payers' increasing orientation toward “pay-for-performance” models evaluated through measures of evidence-based health services delivery and the increased availability of data for clinical decision-making, leaders often described practice change in ways that appealed to their team members' analytical reasoning. Clearly, the need for employee and stakeholder buy-in, which emerged as a theme outside the FRLT model, is a driving force for CHC leaders, given how frequently it was mentioned. Previous research has identified the importance of seeking buy-in from staff as a key component of practice change (38). To get general buy-in, including from the CHC's clinical providers, staff, and external partners and from engaging community members, leaders must communicate confidence that a goal is reachable and the vision is achievable. This exemplifies the inspirational motivation factor of transformational leadership. It may be that seeking buy-in from internal and external stakeholders through actions that fit with the transformational typology generally and inspirational motivation specifically inspire team members to enlist in the mission and share a commitment to serve the CHC patient population. This sets up a dynamic for combining analytical reasoning and creativity to solve difficult problems.

Leaders described using active vigilance to ensure standards are met, a factor of the transactional typology. This was somewhat expected, given the emphasis on adherence to evidence-based guidelines that are built into UDS requirements and affect reimbursement and, consequently, CHC financial viability. Many study participants led CHCs that were moving to an EHR system, and some referenced EHR data as a feedback tool on individual-provider and overall-CHC performance in meeting evidence-based health services delivery goals. Feedback to individual providers positively influences provider behavior and, thus, the quality of care and outcomes (39). The EHR's automatically generated and direct provider feedback could reduce the need for leaders to be as actively vigilant, making the transactional leadership style less relevant for practice change.

Some attributes were not reflected in the data. For example, the transformational typology factor of individualized consideration, that is, leaders paying attention to team members as individuals, was not expressed by participants. Clinical care is often delivered by teams of practitioners, and participants reflected this approach rather than an individualized one. As expected, leaders rarely described the transactional and passive-avoidant typologies.

The CHC is a dynamic environment with many demands on leaders who must plan, prioritize, make trade-off decisions, seek buy-in, and develop resources to advance initiatives. It is unlikely that a passive-avoidant leader, someone who waited until problems arose before acting, would last long at the helm of a CHC.

The 3 new themes emerging from the study–planning, seeking buy-in, and developing resources, may reflect the resource-constrained context in which the participants lead. The theme of buy-in, which occurred frequently, includes buy-in from internal providers and staff as well as external partners. The FRLT describes transformational leadership as leadership that changes those who are members of the team. The process of seeking buy-in from internal and external stakeholders through actions that fit with a transformational typology and, specifically, inspirational motivation, may inspire and motivate team members to enlist in the mission and, through this experience, share a leader's commitment to serving their CHC patient population. However, buy-in from health care providers within the clinic may also require transformational leadership with an emphasis on intellectual stimulation, both in challenging the status quo and appealing to employees' analytical reasoning. This, then, sets up a dynamic to inspire internal stakeholders to buy into the mission and act on that buy-in by combining creativity and analytical reasoning to find solutions to seemingly intractable problems. While the FRLT framework is relevant in this CHC context, it may insufficiently explain the combination of leadership attributes required for leaders' effectiveness in highly resource-constrained health service delivery settings. As described earlier, leadership is a component of a number of organizational processes that influence EBP implementation in clinical settings (21). Understanding the role of leadership more deeply through exploration of the leadership attributes for practice change in the CHC setting adds to the broader understanding of what is required for success.

We based our findings on self-described expressions of leadership, not on objectively observed characteristics. Focus group and interview participants may have perceived a transformational leadership style as preferred and emphasized attributes of that style in their responses, biasing the results toward this typology. Future studies could take this into consideration by comparing the leader's perception of his/her style to that of the employees, as perceptions may vary. Given that this analysis was part of a larger study, there was only one broad leadership question; this may limit the scope of the discussion regarding what steps leaders take to encourage change and how those steps effect practice change. While participants described leadership traits throughout the transcripts, adding a deeper set of questions specifically on leadership could elicit additional information.

Our sample was limited to executive-level leaders who attended primary care organizations' national or regional conferences or training events. The findings, like those in all qualitative studies, are not thought to be generalizable to a broader population, although our study did not gather opinions of leaders from multiple levels of the organization who may have had alternative perspectives on if and how leadership behavior influences practice change. Despite these limitations, our study's strength lies in its deep assessment of leadership for practice change in CHCs and may be particularly useful given the growth CHCs have experienced under the Patient Protection and Affordable Care Act and the prevalence of similar government-funded clinics in other countries.

Future studies could explain leadership's association with and influence on the effectiveness of practice change. Studies could use mixed methods, combine qualitative interviews and FRLT measures or leadership measures for EBP implementation. The resultant data, combined with data on the effectiveness of practice change initiatives within CHCs, the financial performance of CHCs, and patient health outcomes could help further identify the best leadership style in this setting.

Future studies could also explain the interaction of leadership with other variables influencing practice change to understand how leadership works with variables such as intervention, setting, individual and process characteristics. Setting characteristics are especially important to consider in CHCs as the resource constraints and resultant lack of absorptive capacity for change, for example, may overcome leadership as a driver of practice change (40). Finally, future studies could explore the impact of leadership on practice change in non-U.S. settings, building on work of colleagues in other countries (41–44).

## Conclusions

This study reports insights from CHC leaders on how to more effectively lead practice change. Overwhelmingly, CHC leaders viewed transformational leadership and its associated factors as essential for practice change. Thus, this leadership trait, with its associated attributes and behaviors, should be a focus of leadership training and skill building. Future healthcare system changes, including those resulting from national policy and effecting CHCs in particular, could focus on supporting the growth of leaders. Our findings can help inform CHC leader training, particularly for those whose clinics are preparing to implement practice changes or who are struggling with current practice change.

## Data availability statement

The raw data supporting the conclusions of this article will be made available by the authors, without undue reservation.

## Ethics statement

The studies involving human participants were reviewed and approved by University of Texas Health Science Center at Houston. The participants provided their written informed consent to participate in this study.

## Author contributions

All authors listed have made a substantial, direct, and intellectual contribution to the work and approved it for publication.

## Funding

The following cooperative agreements from the Centers for Disease Control and Prevention and the National Cancer Institute provided funding for the Cancer Prevention and Control Research Network Federally Qualified Health Center Qualitative Inquiry Study, through which the data for this study was collected: 1U48-DP-001909, 1U48-DP-001946, 1U48-DP-001924, 1U48-DP-001938-03, 1U48-DP-001936, 1U48-DP-001949-02, 1U48-DP-001911, 1U48-DP-001934, 1U48-DP-001944, 1U48-DP-001944, and 1U48-DP-001903. NH's time was funded by the Cancer Prevention and Research Institute of Texas (RP170259). During the conduct of this research and the preparation of the manuscript, Jennifer Tektiridis was employed by the University of Texas MD Anderson Cancer Center and funded by the Duncan Family Institute for Cancer Prevention and Risk Assessment.

## Conflict of interest

The authors declare that the research was conducted in the absence of any commercial or financial relationships that could be construed as a potential conflict of interest.

## Publisher's note

All claims expressed in this article are solely those of the authors and do not necessarily represent those of their affiliated organizations, or those of the publisher, the editors and the reviewers. Any product that may be evaluated in this article, or claim that may be made by its manufacturer, is not guaranteed or endorsed by the publisher.
